# Heat Shock Protein-Inducing Property of Diarylheptanoid Containing Chalcone Moiety from *Alpinia katsumadai*

**DOI:** 10.3390/molecules22101750

**Published:** 2017-10-17

**Authors:** Joo-Won Nam, Yun-Sil Lee

**Affiliations:** 1 College of Pharmacy, Yeungnam University, Gyeongsan-si, Gyeongsangbukdo 38541, Korea; 2 Graduate School of Pharmaceutical Sciences, College of Pharmacy, Ewha Womans University, Seoul 03760, Korea; yslee0425@ewha.ac.kr

**Keywords:** *Alpinia katsumadai*, diarylheptanoid, chalcone, heat shock protein (HSP), heat shock factor (HSF)

## Abstract

A new diarylheptanoid containing a chalcone moiety, katsumain H (**1**), was isolated from the seeds of *Alpinia katsumadai*. The structure was elucidated using a combination of 1D/2D NMR spectroscopy and mass spectrometry data analysis. The absolute configurations of C-3, C-5, and C-7 in **1** were assigned based on its optical rotation and after comparing its NMR chemical shifts with those of its diastereoisomers, katsumain E and katsumain F, which were previously isolated from this plant and characterized. In this study, the stimulatory effects of compounds **1** and **2** were evaluated on heat shock factor 1 (HSF1), heat shock protein 27 (HSP27), and HSP70. Compounds **1** and **2** increased the expression of HSF1 (1.056- and 1.200-fold, respectively), HSP27 (1.312- and 1.242-fold, respectively), and HSP70 (1.234- and 1.271-fold, respectively), without increased cytotoxicity.

## 1. Introduction

The seeds of *Alpinia katsumadai* (Zingiberaceae) are used for the treatment of gastric disorders in traditional oriental medicine [1,2]. Previous phytochemical investigations of *A. katsumadai* have led to the isolation and identification of various types of diarylheptanoids, flavonoids, stilbenes, and terpenoids [3,4,5,6,7]. Some components of *A. katsumadai* exhibit various bioactivities, such as anti-inflammatory [8], neuroprotective [9], anti-emetic [10], and anti-oxidative [11] effects.

**Figure 1:**
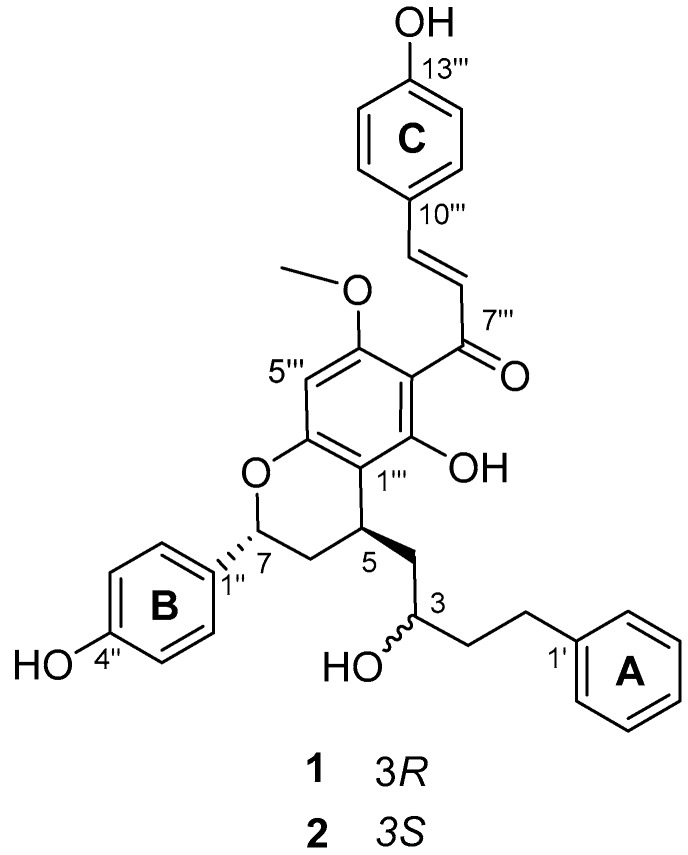
Chemical structures of compounds **1** and **2** from the seeds of *A. katsumadai*.

Heat shock proteins (HSPs) are known to be upregulated in response to stressful conditions, such as hypoxia, inflammation, and infection [12]. HSPs and their key regulator heat shock factor 1 (HSF1) are cytoprotective factors that help restore damaged organs [13]. As part of our continuing search for HSPs, HSF1 inducers, or both from plant sources, we isolated a novel diarylheptanoid compound containing a chalcone moiety from the seeds of *A. katsumadai*. The present paper describes the isolation and elucidation of this new diarylheptanoid, katsumain **H** (**1**), and the induction of HSPs and HSF1 by **1** and its diastereoisomer, katsumain G (**2**) (Figure 1), which was previously isolated and identified [14].

## 2. Results and Discussion

### 2.1. Structure Elucidation of Compound ***1***

**Figure 2:**
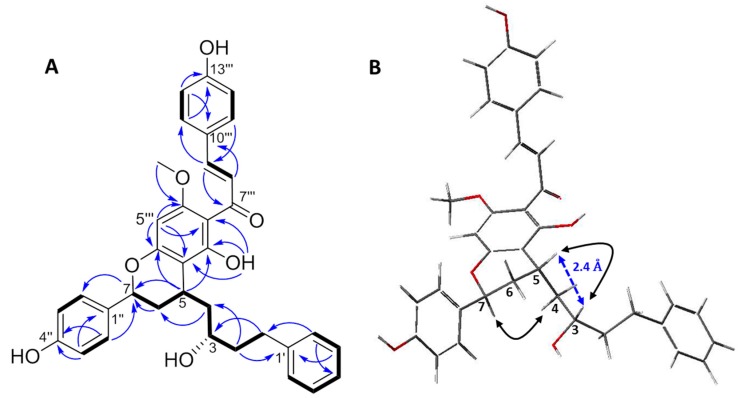
(**A**) Key ^1^H-^13^C HMBC (**→**), ^1^H-^1^H COSY (▬), and (**B**) ^1^H-^1^H NOESY (↔) correlations for compound **1**. **B** is the energy minimized stereostructure of **1** (MM3 calculation using the CAChe 5.0^TM^ molecular modeling program).

Compound **1** was isolated as an amorphous yellow powder, and it showed an ion peak of protonated molecule at *m*/*z* 567.2377 [M + H]^+^ in the high-resolution electrospray ionization mass spectrometry (HRESIMS), which is consistent with an elemental formula of C_35_H_35_O_7_. The ^1^H- and ^13^C-NMR spectra of **1** exhibited resonances for one mono-substituted phenyl ring (A ring: *δ*_H_ 7.23/*δ*_C_ 129.1, 7.23/129.3, and 7.13/126.4, and *δ*_C_ 143.8 and 126.4) and two *p*-substituted phenyl rings (B and C rings: *δ*_H_ 7.63/*δ*_C_ 131.4, 7.33/128.7, 6.93/116.9, and 6.89/116.0, and *δ*_C_ 160.8, 158.2, 133.1, and 128.1). The signals for four methylenes showed at *δ*_H_ 2.88 (H-1a) and 2.71 (H-1b)/*δ*_C_ 32.7 (C-1), 1.83 (H-2)/40.7 (C-2), 2.01 (H-4a) and 1.74 (H-4b)/43.7 (C-4), and 2.30 (H-6a) and 1.97 (H-6b)/35.0 (C-6). Additionally, signals for three methines were also observed at *δ*_H_ 3.84 (H-3)/*δ*_C_ 70.6 (C-3), 3.20 (H-5)/28.3 (C-5), and 5.25 (H-7)/75.5 (C-7). The resonances for the four methylenes and three methines suggested the presence of a diarylheptanoid moiety, which was confirmed by the COSY correlations between the consecutively connected protons, H-1 to H-7 (Figure 2). The A and B rings were positioned at C-1 and C-7, respectively, which were assigned by the heteronuclear multiple-bond correlation (HMBC) spectroscopy cross-peaks of H-2′ (and 6′)/C-1 and H-2′′ (and H-6′′)/C-7. In the ^1^H-NMR spectrum, a singlet at *δ*_H_ 3.98 (3H) was indicative of a methoxyl group, which was positioned at C-4′′′ based on the observed HMBC cross-peaks between the methoxy protons and C-4′′′. Moreover, resonances for a *trans*-olefinic group were observed at *δ*_H_ 7.94 (H-8′′′) and 7.80 (H-9′′′), with a large coupling constant of 15.6 Hz. The ^1^H-NMR spectrum also showed a singlet for a hydrogen-bonded phenolic OH group at *δ*_H_ 15.32. In the ^13^C-NMR spectrum, resonance for the carboxyl group was shown at *δ*_C_ 193.4 (C-7′′′). These observations indicated the presence of a chalcone skeleton. Further analyses of the correlation spectroscopy (COSY), nuclear Overhauser effect spectroscopy (NOESY), heteronuclear single-quantum correlation (HSQC), and HMBC spectra allowed the detailed assignment of the ^1^H and ^13^C resonances (Figure 2). These data suggested that the planar structure of **1** was the same as that of the previously reported katsumain G (**2**), a diarylheptanoid fused with a chalcone moiety [14].

The relative configurations of C-3, C-5, and C-7 in **1** were determined in the same manner as for katsumains E, F, and G (**2**) [14]. The NOESY spectrum showed no correlation between H-5 and H-7, whereas a correlation between H-4 and H-7 was observed, which indicated that H-5 and H-7 were on opposite sides (5R^*^ and 7S^*^, Figure 2). To determine the relative configurations at C-3 and C-5, an energy minimized molecular model was built for **1** with 3S^*^ and 5R^*^ configurations. The interatomic distance between protons H-3 and H-5 was calculated as 2.4 Å (<3 Å), which is expected to lead to NOE correlation [15,16]. A strong NOE cross-peak between H-3 and H-5 was observed, which indicated that the relative configurations of C-3 and C-5 were supposed to be 3S^*^ and 5R^*^, respectively, which were the same as those of katsumain F [14].

**Figure 3:**
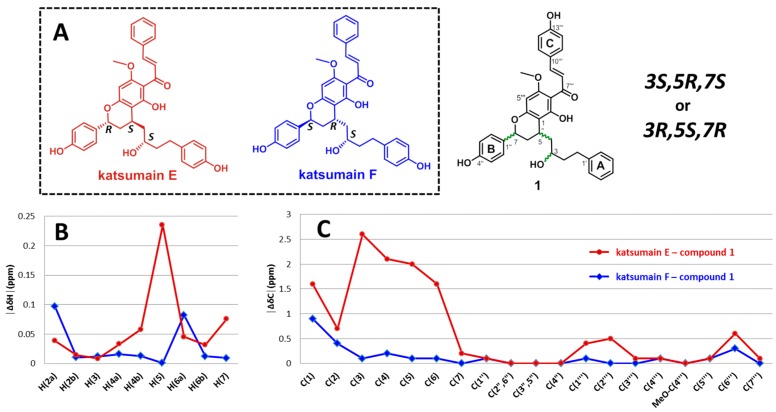
Comparison of ^1^H (**B**) and ^13^C (**C**) chemical shift differences between model compounds (katsumain E and katsumain F; **A**) and compound **1**.

These results were further confirmed by comparing the ^1^H and ^13^C chemical shifts of **1** with those of the model molecules, katsumains E and F (Figure 3A). The relative configurations of C-5 and C-7 were determined to be *trans*, and the number of possible relative configurations for the entire structure is two (3R^*^,5R^*^,7S^*^ or 3S^*^,5R^*^,7S^*^). The structures of katsumains E and F are quite similar to that of compound **1**, except for the hydroxylation patterns of the A and C rings. The relative configurations at C-3, C-5, and C-7 of katsumains E and F are 3R^*^,5R^*^,7S^*^ and 3S^*^,5R^*^,7S^*^, respectively. In the NMR spectra of katsumains E and F, ^1^H-NMR resonances for H-5 to H-7 and ^13^C-NMR resonances for C-3 to C-6 were identified as diagnostic peaks for these two possible diastereoisomers (see Supplementary data), owing to their significant differences in chemical shift (∆δ_H_ and ∆δ_C_ in ppm, │∆δ_H_│_max_ > 0.2 ppm, │∆δ_C_│_max_ > 2.5 ppm). These differences are considered to be a valid difference for the determination of the relative stereochemistry of unknown structures using the Universal NMR database (UDB), a collection of NMR data of partial fragments with possible relative configurations that form the entire polyketide structures [17,18,19]. To estimate the similarities between **1** and each of the model compounds, katsumains E and F, the absolute chemical shift differences (│∆δ│ [ppm]) were calculated. As shown in Figure 3B,C, the absolute chemical shift differences at the diagnostic points (H-5 to H-7 and C-3 to C-6) between katsumain F and **1** were smaller than those between katsumain E and **1**. Furthermore, this evaluation supported the suggestion that the relative configurations of **1** were more similar to those of katsumain F (3S^*^,5R^*^,7S^*^) than they were to those of katsumain E (3R^*^,5R^*^,7S^*^). This indicated that the relative configurations of **1** are 3S^*^,5R^*^,7S^*^.

The absolute configurations of **1** were determined by comparing its optical rotations with those of katsumain F (3*S*,5*R*,7*S*) and a synthetic analog, calyxin F (3*S*,5*R*,7*S*). The optical rotations of katsumain F and calyxin F were reported as ${\left[\alpha \right]}_{D}^{25}$ = +15.91 (*c* 0.1, MeOH) and ${\left[\alpha \right]}_{D}^{25}$ = +16.3 (*c* 0.175, MeOH), respectively [14,20]. Thus, the absolute configurations at C-3, C-5, and C-7 of **1** were determined as 3*R*,5*S*,7*R* owing to the opposite sign of the optical rotation (${\left[\alpha \right]}_{D}^{25}$ = −4.81, *c* 0.1, MeOH).

The results indicate that the structure of **1** was most compatible with that of a new diarylheptanoid, (2*E*)-1-{(2*R*,4*S*)-3,4-dihydro-5-hydroxy-4-[(2*R*)-2-hydroxy-4-phenyl-butyl]-2-(4-hydroxy-phenyl)-7-methoxy-2*H*-1-benzopyran-6-yl}-3-(4-hydroxyphenyl)-2-propen-1-one, namely, katsumain H.

### 2.2. Induction of HSF1 and HSPs by Compounds ***1*** and ***2***

The inductive effects of compounds **1** and **2** on HSF1 and its transcriptional targets, HSP27 and HSP70, were evaluated using western blotting in NCI-H460 cells collected after a 24-h treatment with the isolated compounds. Celastrol, an HSP inducer, was used as a positive control that did not affect HSF1 expression. As shown in Table 1, compounds **1** and **2** increased the expression of both HSP27 and HSP70, whereas only compound **2** increased HSF1 expression. These results indicate that the mechanism of compound **2** differed from that of celastrol. Moreover, compounds **1** and **2** did not show any cytotoxicity against NCI-H460 cells (IC_50_ > 30 μM). In conclusion, **1** and **2** could be potential leads for development as cytoprotective agents for the treatment of damaged organs through the induction of HSF1, HSPs, or both.

## 3. Materials and Methods

### 3.1. General Procedures

Optical rotation was measured using a P-1010 polarimeter (Jasco, Tokyo, Japan) at 25 °C. UV spectrum was recorded using a U-3000 spectrophotometer (Hitachi, Tokyo, Japan). 1D and 2D NMR experiments were performed using the UNITY INOVA 400 MHz FT-NMR instrument (Varian, Palo Alto, CA, USA), with chemical shifts given in ppm (*δ*). Tetramethylsilane (TMS) (Merck, Darmstadt, Germany) was used as an internal standard. Mass spectrometry was recorded using a Waters ACQUITY UPLC system (Waters, Milford, MA, USA) coupled to a Micromass Q-Tof Micro mass spectrometer and an Agilent 6220 Accurate-Mass TOF LC/MS system (Agilent, Santa Clara, CA, USA). Silica gel (230–400 mesh, Merck, Darmstadt, Germany) and Sephadex LH-20 (GE Healthcare, Uppsala, Sweden) were used for the column chromatography (CC). Thin layer chromatography (TLC) was performed using Kieselgel 60 F 254 (silica gel, 0.25 mm layer thickness, Merck, Darmstadt, Germany) and RP-18 F 254s (Merck, Darmstadt, Germany) plates, with visualization under UV light (254 and 365 nm) and 10% (*v*/*v*) H_2_SO_4_ spray followed by heating (120 °C, 5 min). Preparative HPLC was carried out using an Acme 9000 system (Young Lin, Anyang, Korea) using YMC J’sphere ODS-H80 (4 μm, 250 × 20 mm i.d.) column.

### 3.2. Plant Material

The seeds of *A. katsumadai* were purchased from the Kyungdong Oriental Herbal market in Seoul, South Korea (May 2010), and were identified by Professor Je-Hyun Lee (College of Oriental Medicine, Dongguk University). A voucher specimen (no. EA299) was deposited at the Natural Product Chemistry Laboratory, College of Pharmacy, Ewha Womans University.

### 3.3. Extraction and Isolation

The extraction methods of *A. katsumadai* seeds and the preparation of fractions Fr.11.1–Fr.11.17 were described in a previous report [14]. Briefly, the dried seeds were extracted with MeOH at room temperature. After drying under vacuum, the MeOH extract (788 g) was subjected to solvent partitioning with hexane, EtOAc, and BuOH, sequentially. The EtOAc extract (150 g) was applied to silica gel CC to obtain 16 subfractions, Fr.01–Fr.16. Fr. 11 was chromatographed on reversed-phase CC to yield 17 subfractions, Fr.11.01–Fr.11.17. Among the fractions, Fr.11.14 (0.520 g) was chosen and chromatographed using a silica gel column (60 g) with gradient mixtures of hexanes–EtOAc (9:1→7:3), resulting in seven subfractions, Fr.11.14.01–Fr.11.14.07. Fr. 11.14.03 was subjected to HPLC (YMC J’sphere ODS-H80, 4 μm, 250 × 20 mm i.d.) using an isocratic mixture of MeOH-0.1% formic acid in water (87:13, 2 mL/min) to obtain four subfractions (Fr.11.14.03.01–Fr.11.14.03.04). Fr.11.14.03.03 was further purified using Sephadex LH-20 with 100% MeOH to afford katsumain H (**1**, 8.8 mg, 0.00016% *w*/*w*). Compound **1** was detected as a yellowish spot after heating with 10% (*v*/*v*) sulfuric acid spray on a reversed-phase TLC developed with a MeOH–water mixture (5:5, *R*_f_ 0.37).

*Katsumain H* (**1**). Yellow amorphous powder; ${\left[\alpha \right]}_{D}^{25}$ −4.81 (*c* 0.2, MeOH); UV (MeOH) λ_max_ (log ε) 372 (4.7), 227 (4.7) nm; ^1^H- and ^13^C-NMR, see Table 2; HMBC correlations H-4′/C-2′, C-3′, C-5′, C-6′; H-3′ and H-5′/C-1′; H-2′ and H-6′/C-3′, C-4′, C-5′, C-1; H-1/C-2, C-3, C-1′, C-2′, C-6′; H-2/C-1′, C-1, C-3; H-3/C-2, C-4; H-4/C-2, C-3, C-5, C-6; H-5/C-3, C-4, C-7, C-2′′′, C-6′′′; H-6/C-4, C-5, C-7, C-1′′; H-7/C-5, C-6, C-1′′, C-2′′, C-6′′; H-2′′ and H-6′′/C-7, C-3′′, C-4′′, C-5′′; H-3′′ and H-5′′/C-1′′, C-4′′; H-5′′′/C-5, C-1′′′, C-3′′′, C-4′′′, C-6′′′, C-7′′′; OCH_3_-4′′′/C-4′′′; OH-2′′′/C-1′′′, C-2′′′, C-3′′′, C-7′′′; H-8′′′/C-7′′′, C-9′′′, C-10′′′; H-9′′′/C-7′′′, C-8′′′, C-10′′′, C-11′′′, C-15′′′; H-11′′′ and H-15′′′/C-9′′′, C-12′′′, C-13′′′, C-14′′′; H-12′′′ and H-14′′′/C-10′′′, C-13′′′; NOESY correlations H-3/H-5, H-5/H-6b, H-7/H-6a, H-1/H-2′ and H-6′, H-6/H-2′′ and H-6′′, H-7/H-2′′ and H-6′′, OCH3-4′′′/H-5′′′, H-8′′′/H-11′′′ and H-15′′′, H-9′′′/H-11′′′ and H-15′′′; HRESIMS *m*/*z* 567.2377 [M + H]^+^ (calcd. for C_35_H_35_O_7_, 567.2383).

### 3.4. Western Blot Analysis

The modulatory effects of compounds **1** and **2** on HSF1 and the HSPs’ expression were evaluated using a previously established protocol [21]. Proteins in lysates were separated by SDS-PAGE, electrotransferred to nitrocellulose membranes (GE Healthcare, Amershan, UK), subsequently blotted with specific antibodies, and visualized using an ECL detection system (Thermo Scientific, Waltham, MA, USA). Anti-HSF1, anti-Hsp27, anti-Hsp70, and *β*-actin antibodies were purchased from Santa Cruz Biotechnology (California, CA, USA). All of the results represent the mean ± SD of three independent experiments performed in triplicate 24 h after treatment. Furthermore, *p*-values < 0.05 were considered statistically significant, comparing the quantitative values of HSP70, HSP27, or HSF1 expression levels between treated and untreated control cells.

### 3.5. MTT Assay

The cells were tested for their cytotoxicity in the 3-(4,5-dimethylthiazol-2-yl)-2,5-diphenyltetrazoliumbromide (MTT; Sigma, St. Louis, MO, USA) test, as previously described [22].

## Figures and Tables

**Table 1:** Induction of heat shock factor (HSF) 1 and heat shock proteins (HSPs) by compounds **1** and **2** isolated from *A. katsumadai*.

Compound	Fold Increase ^a^			IC_50_ (μM) ^b^
	HSF1	HSP27	HSP70	
**1**	1.056 ± 0.023	1.312 ± 0.013	1.234 ± 0.016	44.1
**2**	1.200 ± 0.030	1.242 ± 0.016	1.271 ± 0.026	42.1
Celastrol ^c^	1.066 ± 0.009	1.216 ± 0.022	1.371 ± 0.037	12.3
Taxol ^d^	ND ^e^	ND ^e^	ND ^e^	8.0

^a^ Summary of quantitative immunoblotting results of HSF1, HSP27, and HSP70 in human non-small cell lung cancer (NCI-H460) cells after normalization to *β*-actin signal. ^b^ IC_50_ values were the concentrations (μM) of 50% inhibition of cell growth in NCI-H460 cells. ^c^ Celastrol was used as the positive control for HSP expression. ^d^ Taxol was used as a positive control for cytotoxicity. ^e^ ND; not detected.

**Table 2:** ^1^H-(400 MHz) and ^13^C-(100 MHz) NMR data for compound **1**.

Position	1	
	*δ*_H_	*δ*_C_
1	2.71 m 2.88 m	32.7
2	1.83 m	40.7
3	3.84 m	70.6
4	1.74 m	43.7
	2.01 m	
5	3.20 m	28.3
6	1.97 dd (13.8, 5.2)	35.0
	2.30 d (13.8)	
7	5.25 dd (12.4, 1.6)	75.5
1′		143.8
2′,6′	7.23 m	129.1
3′,5′	7.23 m	129.3
4′	7.13 m	126.4
1′′		133.1
2′′,6′′	7.33 d (8.4)	128.7
3′′,5′′	6.89 d (8.4)	116.0
4′′		158.2
1′′′		108.0
2′′′		166.5
3′′′		106.3
4′′′		161.9
5′′′	6.05 s	92.6
6′′′		162.6
7′′′		193.4
8′′′	7.94 d (15.6)	125.2
9′′′	7.80 d (15.6)	143.7
10′′′		128.1
11′′′,15′′′	7.63 d (8.8)	131.4
12′′′,14′′′	6.93d (8.8)	116.9
13′′′		160.8
OCH_3_-4′′′	3.98 s	56.4
OH-2′′′	15.32 s	

Chemical shifts (*δ*) are expressed in ppm, *J* values are in parentheses. Data were measured in acetone-*d*_6_.

## Supplementary Materials

Supplementary materials are available online.
