# Supplementary material for: Heat Shock Protein-Inducing Property of Diarylheptanoid Containing Chalcone Moiety from Alpinia katsumadai
Source: Molecules. 2017 Oct 17;22(10):1750. doi: 10.3390/molecules22101750 (PMC6151646; doi:10.3390/molecules22101750)
Supplement: Supplementary file 1 [file molecules-22-01750-s001.pdf]

Supplementary data

Heat Shock Protein-Inducing Property of Diarylheptanoid-Containing Chalcone Moiety from *Alpinia katsumadai*

Joo-Won Nam[a,*] and Yun-Sil Lee[b]

[a] *College of Pharmacy, Yeungnam University, Gyeongsan-si, Gyeongsangbukdo 38541, Korea*

[b] *Graduate School of Pharmaceutical Sciences, College of Pharmacy, Ewha Womans University, Seoul 03760, Korea*

* Corresponding author: e-mail address: jwnam@yu.ac.kr (J.-W. Nam)

Figure	S1.	^{1}H NMR (400 MHz, acetone-d_6) of katsumain H (**1**) ···································	2
Figure	S2.	^{13}C NMR (100 MHz, acetone-d_6) of katsumain H (**1**) ·································	3
Figure	S3.	^{1}H-^{1}H COSY NMR (acetone-d_6) of katsumain H (**1**) ······························	4
Figure	S4.	^{1}H-^{1}H NOESY NMR (acetone-d_6) of katsumain H (**1**) ····························	5
Figure	S5.	^{1}H-^{13}C HSQC NMR (acetone-d_6) of katsumain H (**1**) ····························	6
Figure	S6.	^{1}H-^{13}C HMBC NMR (acetone-d_6) of katsumain H (**1**) ····························	7
Figure	S7.	Graphical representation of the chemical shift differences between the model compounds, katsumain E and katsumain F ·····································	8
Figure	S8.	Western blot analysis ··	9

Figure S1. ^{1}H NMR (400 MHz, acetone-d_6) of katsumain H (**1**)

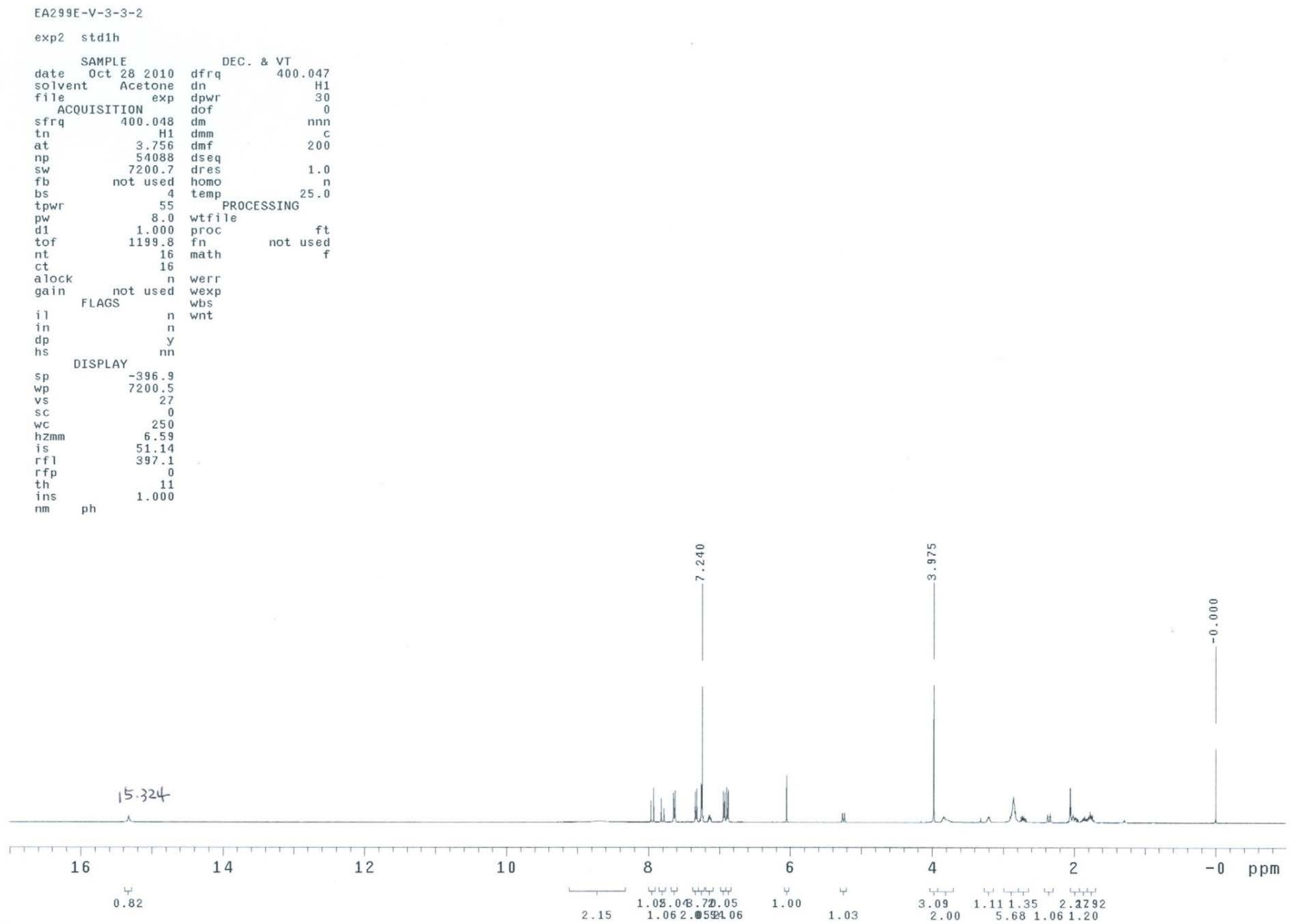

Figure S2. ^{13}C NMR (100 MHz, acetone-d_6) of katsumain H (**1**)

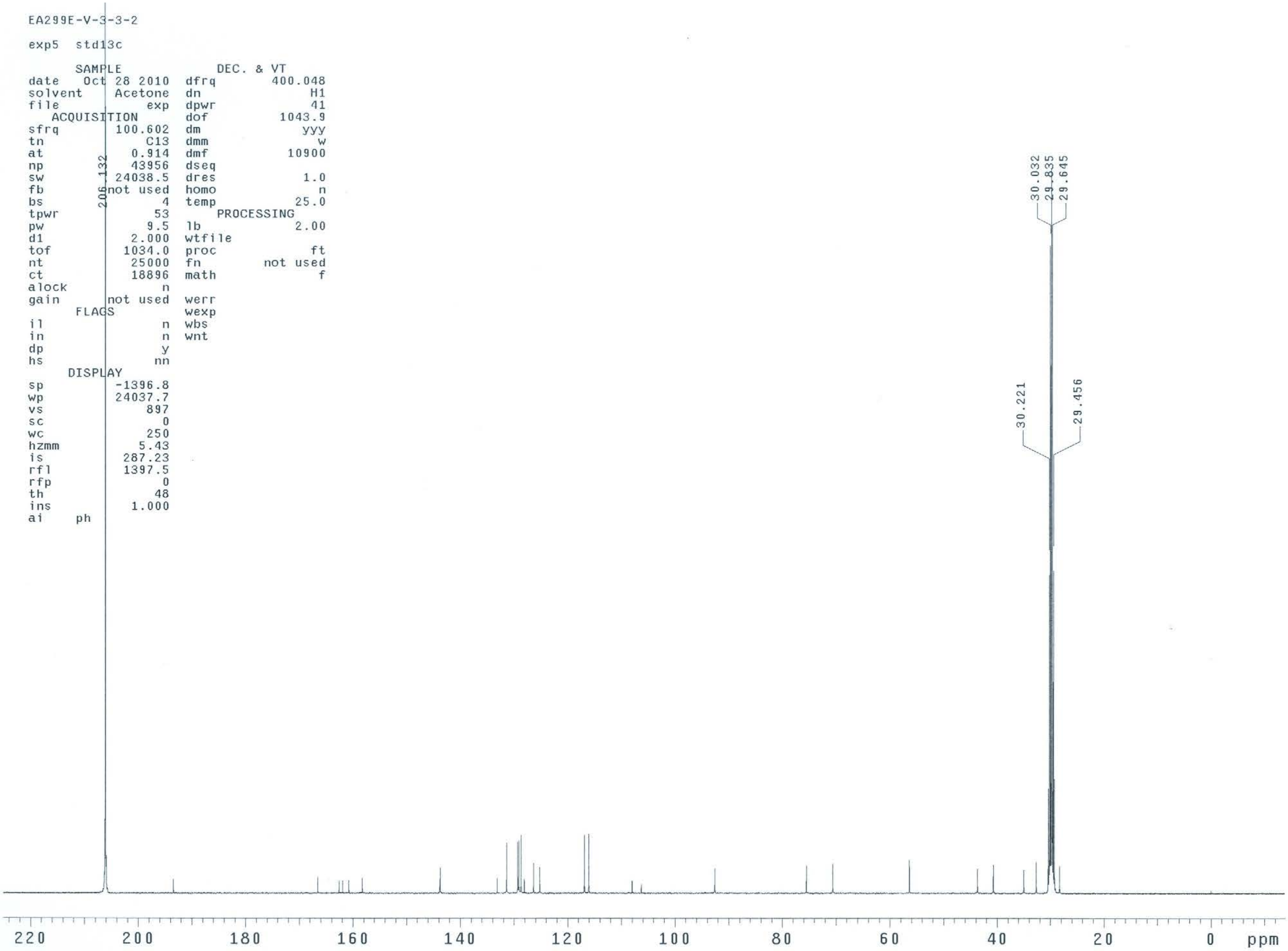

Figure S3. ^{1}H-^{1}H COSY NMR (acetone-d_6) of katsumain H (**1**)

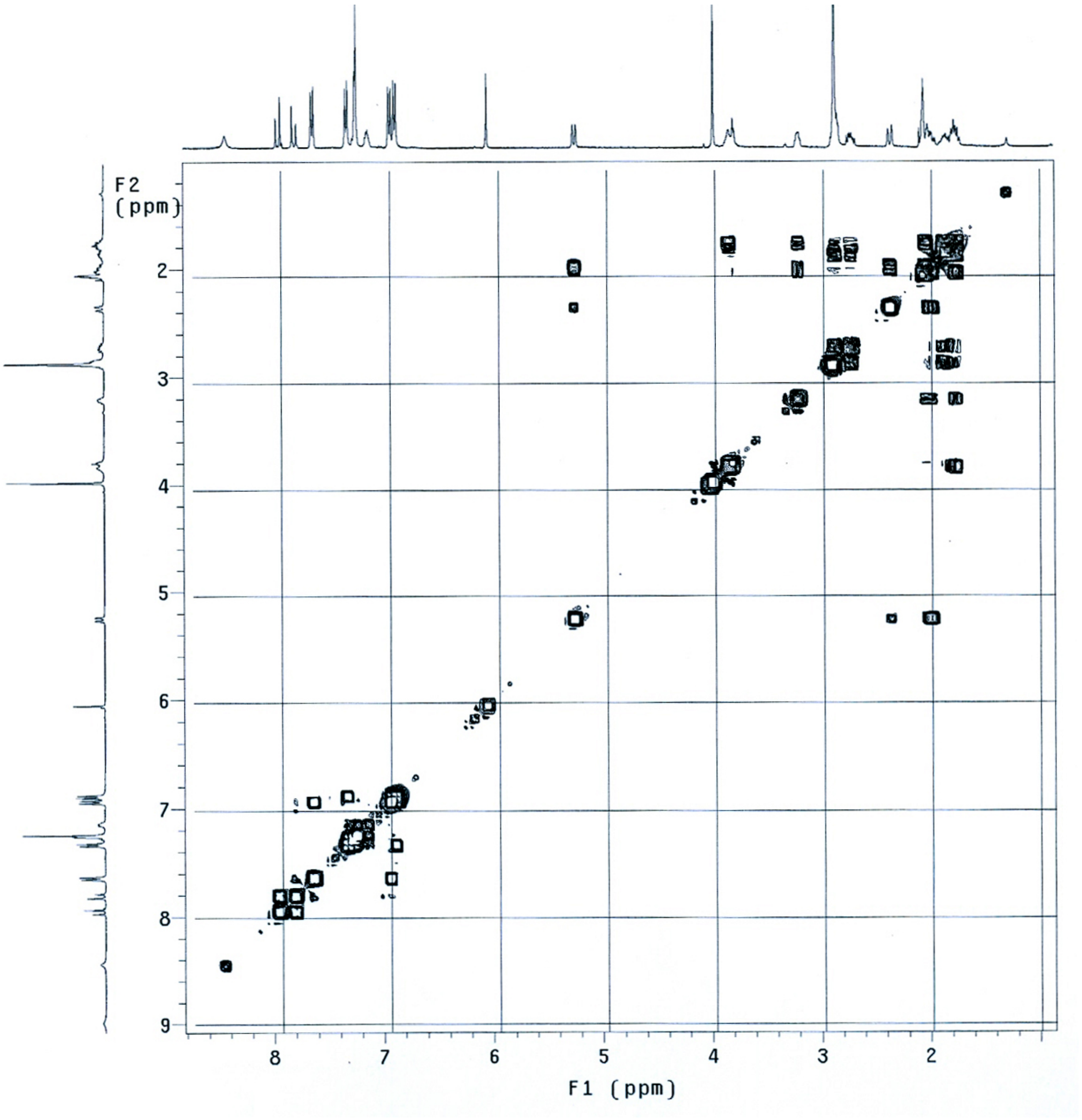

Figure S4. ^{1}H-^{1}H NOESY NMR (acetone-d_6) of katsumain H (**1**)

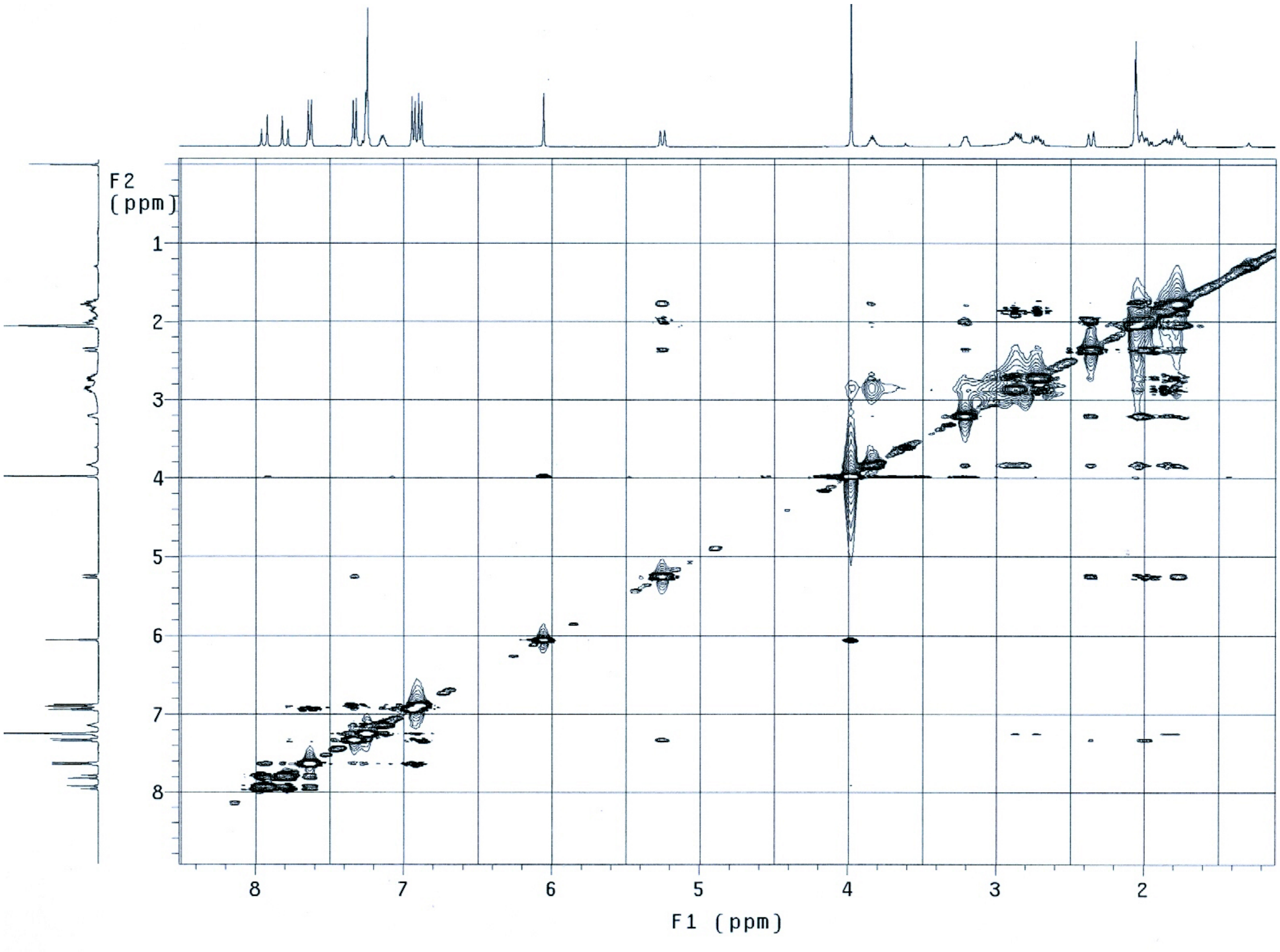

Figure S5. ^{1}H-^{13}C HSQC NMR (acetone-d_6) of katsumain H (**1**)

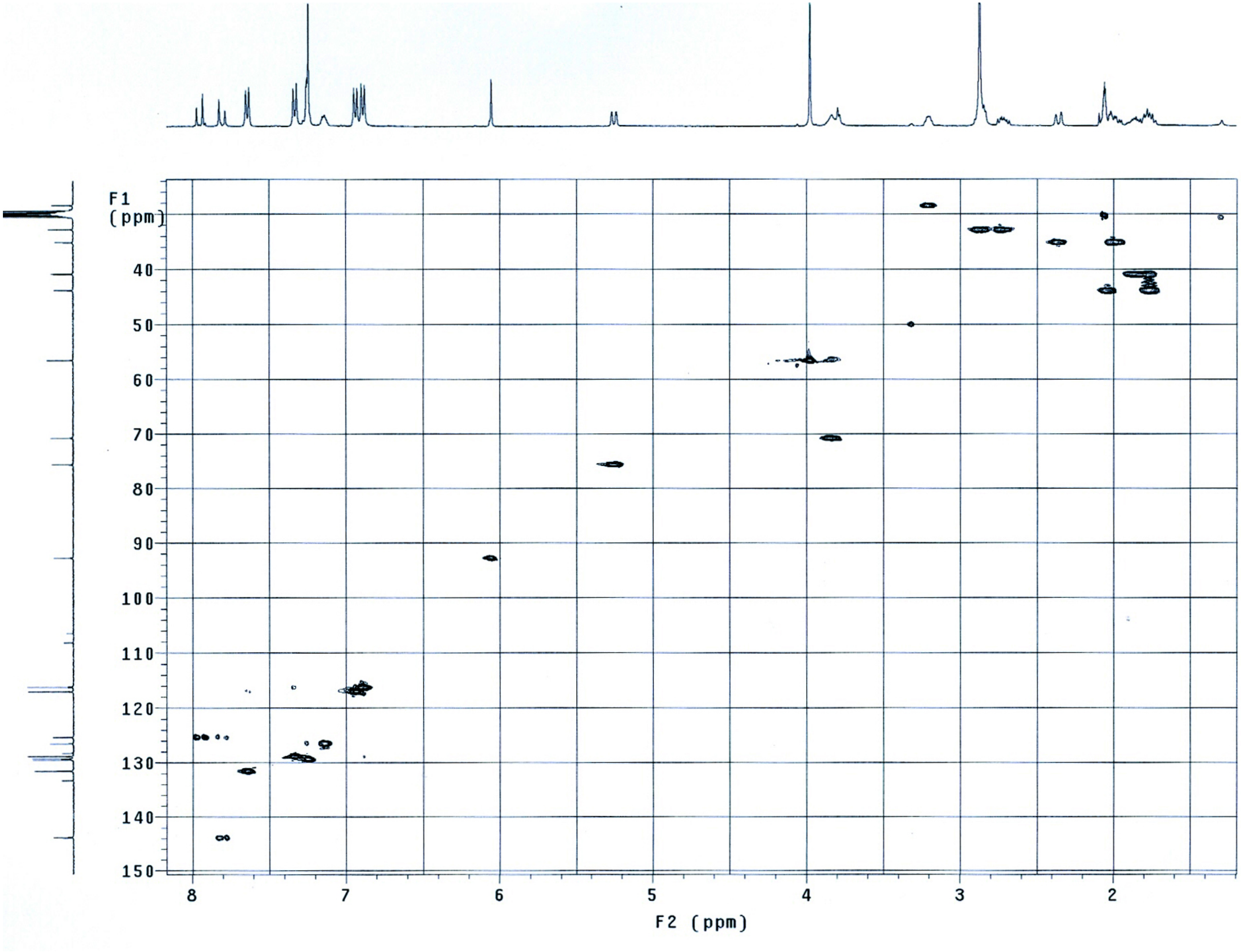

Figure S6. ^{1}H-^{13}C HMBC NMR (acetone-d_6) of katsumain H (**1**)

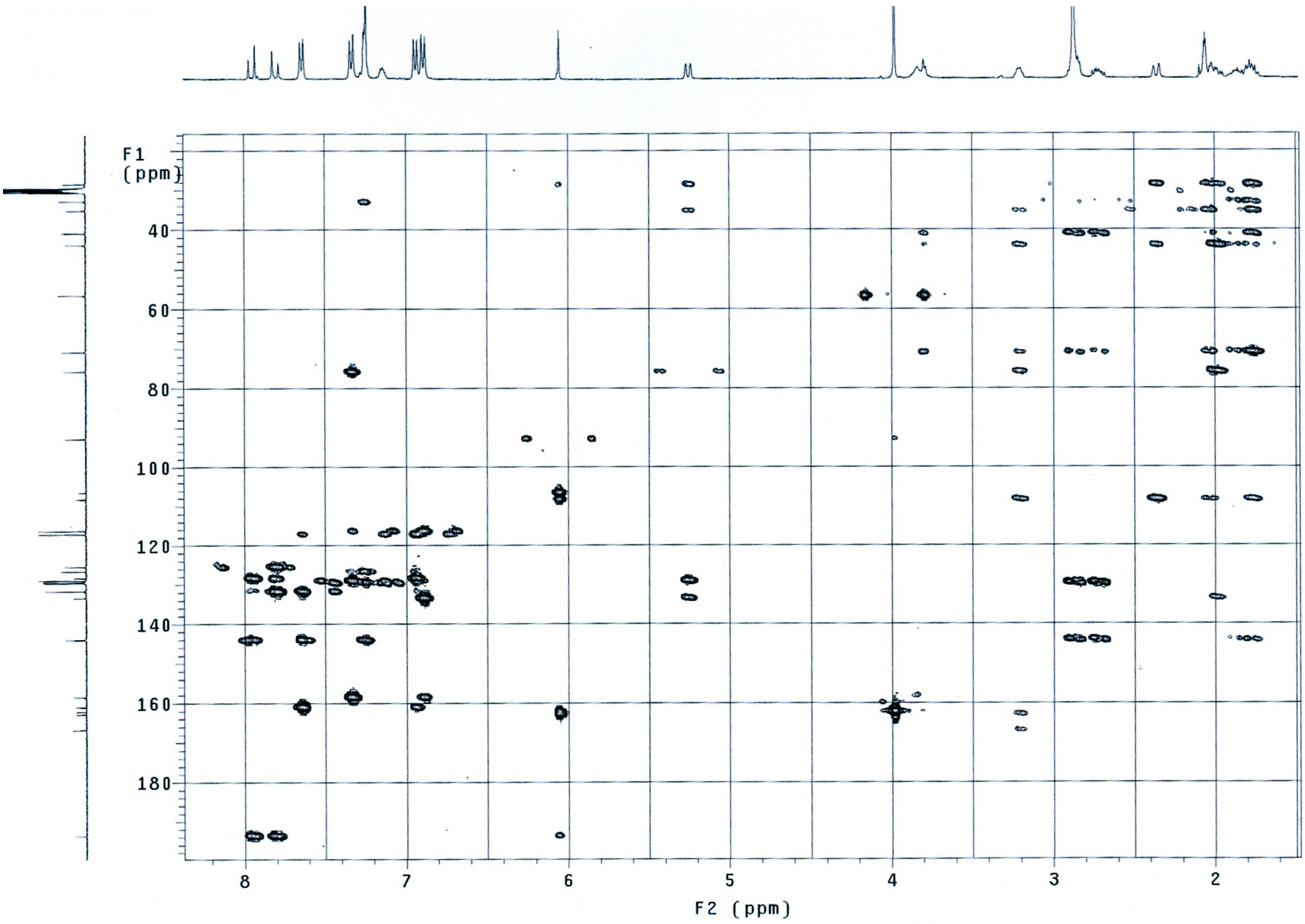

Figure S7. Graphical representation of the chemical shift differences (A: $\Delta\delta_H$ [ppm]; B: $\Delta\delta c$ [ppm]) between the model compounds, katsumain E and katsumain F (acetone-d_6, 600 MHz and 150 MHz for ^1H and ^{13}C, respectively) [14].

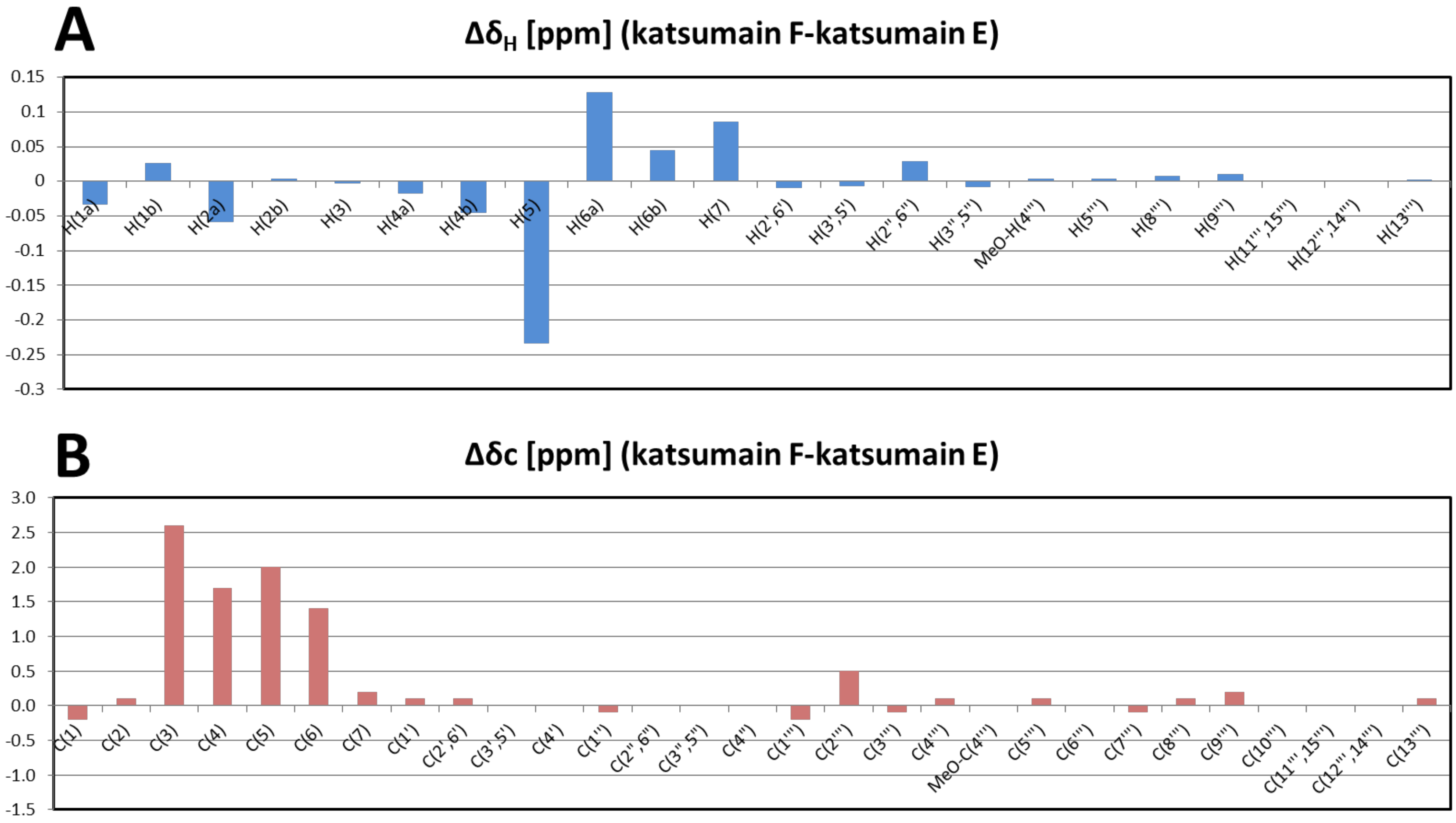

Figure S8. Western blot analysis

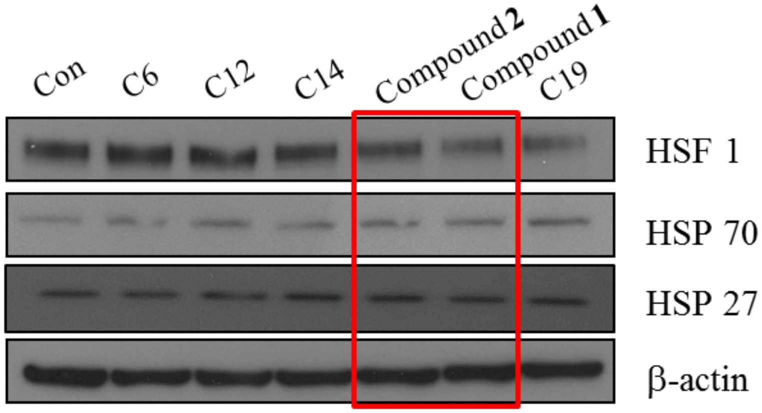

Western blot analysis was performed 24 hours after sample treatment. The results for C6, C12, C14, and C19 are not mentioned in this paper.
